# Clinical Analysis of Pediatric Systemic Juvenile Xanthogranulomas: A Retrospective Single-Center Study

**DOI:** 10.3389/fped.2021.672547

**Published:** 2021-06-10

**Authors:** Hongyun Lian, Ang Wei, Lejian He, Ying Yang, Honghao Ma, Liping Zhang, Yitong Guan, Qing Zhang, Dong Wang, Zhigang Li, Rui Zhang, Tianyou Wang

**Affiliations:** ^1^Hematology Center, Beijing Key Laboratory of Pediatric Hematology Oncology, Beijing, China; ^2^National Key Discipline of Pediatrics, Capital Medical University, Beijing, China; ^3^Key Laboratory of Major Disease in Children, Ministry of Education, Beijing, China; ^4^Beijing Children's Hospital, Capital Medical University, National Center for Children's Health, Beijing, China; ^5^Department of Pathology, Beijing Children's Hospital Affiliated With Capital Medical University, National Center for Children's Health, Beijing, China; ^6^Hematologic Disease Laboratory, Hematology Center, Beijing Key Laboratory of Pediatric Hematology Oncology, Beijing, China; ^7^Beijing Pediatric Research Institute, Beijing Children's Hospital, Capital Medical University, National Center for Children's Health, Beijing, China

**Keywords:** juvenile xanthogranuloma, clinical characteristics, treatment, prognosis, system

## Abstract

**Objective:** To investigate the clinical characteristics, treatment, and prognosis of children with systemic juvenile xanthogranuloma (JXG).

**Methods:** Clinical data of children with JXG who were hospitalized in Beijing Children's Hospital, Capital Medical University, from January 2012 to December 2019 were retrospectively analyzed, including clinical manifestations, laboratory determinations, treatment, and prognosis of the children. Patients were treated with vindesine + prednisone as the first-line treatment and cytarabine + vindesine + dexamethasone ± cladribine as the second-line treatment.

**Results:** Ten patients, including 8 males and 2 females, with a median of onset age of 1.95 (0.80–7.30) years, exhibited multi-system dysfunction. The median age of diagnosis was 2.45 (1.30–12.10) years. The most common location of extracutaneous lesions was the central nervous system (6 cases), followed by the lung (5 cases) and bone (4 cases). Nine patients underwent first-line chemotherapy, and 6 patients underwent second-line chemotherapy, including 5 patients with poorly controlled disease after first-line treatment. The median observation time was 29 (3–115) months. Nine patients survived, whereas one patient died of respiratory failure caused by pulmonary infection. At the end of follow-up, 7 patients were in active disease (AD)/regression state (AD-better), and 2 patients were in an AD/stable state (AD-stable). Three patients had permanent sequelae, mainly central diabetes insipidus. The rates of response to the first-line treatment and the second-line treatment were 40.0 and 66.7% respectively.

**Conclusion:** The chemotherapy protocol for Langerhans cell histiocytosis (LCH) may be effective for patients with systemic JXG. Central nervous system involvement may not impact overall survival, but serious permanent sequelae may occur.

## Introduction

Juvenile xanthogranuloma (JXG) is the most common non-Langerhans cell histiocytosis in children, and the incidence rate is ~1/1,000,000. Based on the different organs involved, it can be classified as cutaneous JXG and systemic (extracutaneous) JXG (1, 2). The most common presentation of JXG is a solitary cutaneous lesion. This type is self-limiting, and skin lesions can resolve spontaneously without treatment. Systemic JXG is rare, accounting for 4–10% of JXG cases. Cases with visceral involvement or intracranial lesions are extremely rare. Although a subset of systemic JXG can also be self-limiting, lesions occurred in eye or central nervous system possibly predict poor prognosis and sequelae (3, 4). The treatment outcome, including late sequelae, of patients with systemic JXG remains unclear due to the rareness of the disease. Here, we report the clinical features and treatment outcome of 10 cases of pediatric patients with systemic JXG.

## Patients and Methods

### Patients

All children suffering from systemic JXG between January 2012 and December 2019 were enrolled in this study. Data were retrospectively reviewed including clinical manifestations, laboratory findings, age at onset, treatment, and outcome. This study was conducted in accordance with the Declaration of Helsinki and approved by the Institutional Review Board (IRB) of Beijing Children's Hospital, Capital Medical University (2020-z-134). All patients and/or their parents or guardians signed informed consent.

### Diagnostic Criteria

The diagnostic criteria for JXG in this study were as follows: characteristic Touton cells (rosette-like nuclei in the center and eosinophilic or vacuolar cytoplasm) observed in biopsy, or Factor XIIIa, CD68, and/or CD163-positive and Langerin, CD1a-negative cells in immunohistochemical staining (5, 6).

BRAF mutations were determined by DNA-based studies and/or immunohistochemistry with a BRAF V600E (VE1) monoclonal antibody.

### Chemotherapy Regimens

Until now, there has been no generally accepted chemotherapy regimen for systemic JXG. Because both JXG and LCH are histiocytic diseases, our center treats systemic JXG with a modified regimen based on the LCH-III and LCHS 2005 of the International Histiocyte Society (6). The details of the chemotherapy regimens are shown below. Due to unavailability of vinblastine in our country, we used vindesine (VDS) instead in our regimens.

#### First-Line Therapy

There are three elements in the first-line therapy: (1) initial induction 1: VDS, 3 mg/m^2^/dose, bolus infusion, once a week for 6 weeks and prednisone (40 mg/m^2^/day) administered orally at two divided doses on days 1–28, followed by gradual tapering for the following 2 weeks; (2) initial induction 2: VDS, 3 mg/m^2^/dose, bolus infusion, once a week for 6 weeks and prednisone (40 mg/m^2^/day) administered orally at two divided doses on days 1–3, weekly for 6 weeks; (3) maintenance: VDS, 3 mg/m^2^/dose, bolus infusion, day 1 every 3 weeks; prednisone, 40 mg/m^2^/day, orally, at two divided doses on days 1–5 every 3 weeks; 6-mercaptopurine (6-MP), 50 mg/m^2^/day, orally, every night. The total duration of the course was 25 or 52 weeks.

#### Second-Line Therapy

Second-line therapy was initiated due to disease progression uncontrolled by the first-line therapy, or as the initial treatment for patients with severe conditions (such as liver, spleen, hematopoietic system, brain and lung involvement). The second-line therapy included two arms.

Arm A (Cytarabine + VDS + dexamethasone): cytarabine, 100 mg/m^2^/day, intravenous guttae within 1 h, days 1–5; VDS, 3 mg/m^2^/dose, bolus infusion, day 1; and dexamethasone, 6 mg/m^2^/day, infusion/oral, days 1–5. There were 21 days per cycle. After 8 cycles, the maintenance treatment was started.

Arm B (Cytarabine + VDS + dexamethasone + cladribine): cytarabine, 100 mg/m^2^/day, intravenous guttae within 1 h, days 1–5; VDS, 3 mg/m^2^/dose, bolus infusion, day 1; dexamethasone, 6 mg/m^2^/day, infusion/oral, days 1–5; and cladribine, 5 mg/m^2^/day, intravenous guttae within 1 h, days 2–6. There was 28 days per cycle. After 4 cycles, 4 cycles of therapy in arm A was used before entering the maintenance treatment.

Maintenance of the second-line therapy was the same as that of the first-line treatment. The total duration of the second-line treatment was 1 year.

### Evaluation of Disease State and Treatment Response

Evaluations were performed at the 5th, 11th, 25th, and 52nd week during the first-line chemotherapy. During the second-line treatment, evaluations were performed every 4 cycles of treatments. During the maintenance treatment, evaluations were carried out every 3 months. Evaluations were also carried out 3 months, 6 months, 1 year, 2 years, 3 years, and 5 years after the end of treatment.

Evaluation items included blood routine and biochemical tests, thyroid function, urine osmotic pressure tests, and imaging examinations (radiography, ultrasound, computed tomography, and/or magnetic resonance imaging of the involved location).

Evaluation was performed according to Histiocyte Society Evaluation and Treatment Guidelines 2009 for LCH (7, 8). Briefly, the disease states included non-active disease (NAD) and active disease (AD). The treatment response was categorized as complete resolution (NAD), regression (AD-Better, AD-B), mixed (AD-Intermediate, AD-I), stable (AD-Stable, AD-S), and progression (AD-Worse, AD-W). The response rate (RR) was defined as the percentage of patients with NAD and AD-B among all patients. Event was defined as death due to any reasons and disease progression or recurrence.

### Statistical Analysis

Statistical analysis was performed by IBM SPSS Statistics 24 software (IBM, USA). Normal distribution data were presented as the median (range).

## Results

### General Information of the Patients

Ten children with systemic JXG were enrolled in this study, including 8 males and 2 females. The ratio of males to females was 4:1. The median age of disease onset was 1.95 (0.80–7.30) years. The median age of diagnosis was 2.45 (1.30–12.10) years. It took a long time to make a diagnosis of systemic JXG in the 3 children with central diabetes insipidus since disease onset with the longest interval of 7 years (Table 1).

**Table 1:** General information.

**Pt**	**Onset age (years)**	**Diagnosis age (years)**	**Sex**	**Biopsy sites**	**Involved location**				
					**Skin**	**CNS**	**Lung**	**Liver**	**Others**
1	0.8	1.5	F	Skin	+	Pituitary gland	+	-	-
2	5.7	6.0	M	Testis	-	-	+	+	Kidney, eye, parotid gland, salivary gland, testis
3	1.4	1.5	M	Skin + liver	+	-	-	+	Blood, spleen
4	2.2	2.5	M	Femur	-	Multiple	-	-	Femur, vertebra
5	7.3	7.3	M	Skin	+	Multiple	+	-	Eye, parotid gland, pancreas, testis
6	1.1	1.4	M	Skin	+	-	+	-	Bone
7	1.2	1.3	F	Femur	-	-	-	-	Thyroid gland, bone
8	1.7	2.4	M	Skin + liver +bone marrow	+	Multiple	+	+	Bone marrow, spleen, bone
9	7.2	7.8	M	Muscle	-	Multiple	-	-	Soft tissue, muscle
10	5.1	12.1	M	Skin + epencephalon	+	Pituitary gland + multiple	-	-	Eye, blood

*Pt, patient; F, female; M, male; CNS, central nervous system*.

### Clinical Manifestations

**Figure 1:**
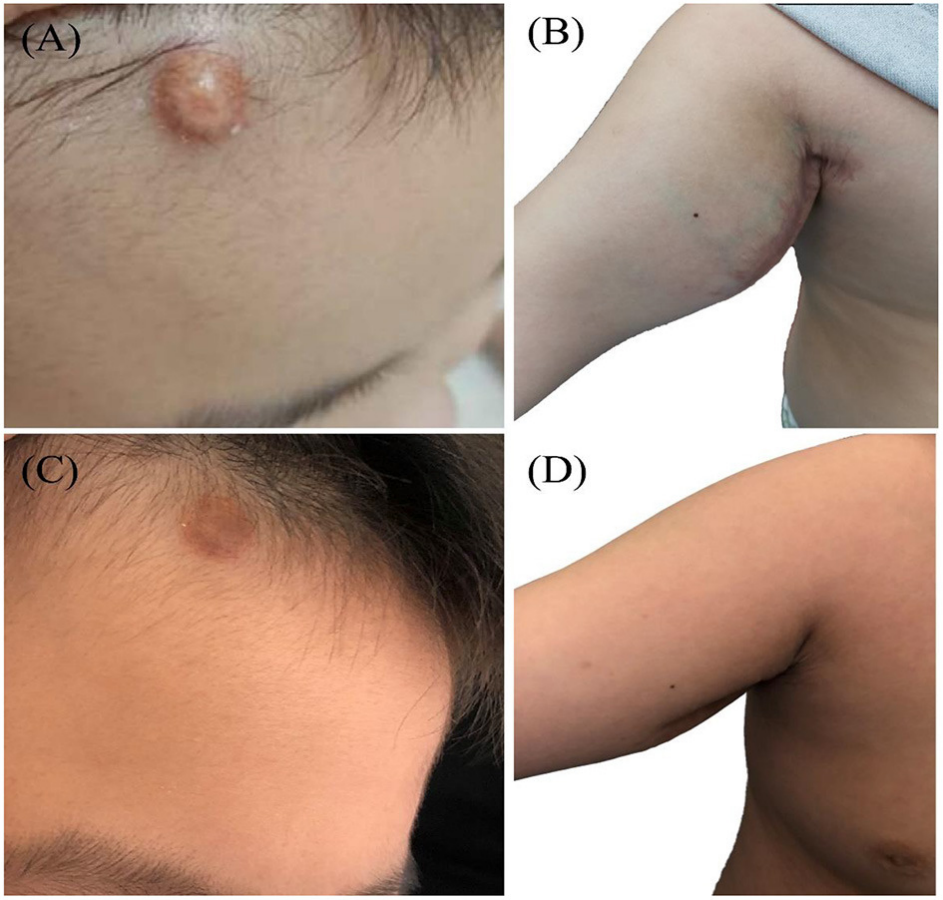
JXG soft tissue mass (case 9): a round mass in the head **(A)** and right axilla **(B)** of the onset, tough, no tenderness. **(C,D)** Showed the mass after treatment.

Various clinical manifestations were observed in the enrolled patients. In the early stage of the disease, subcutaneous masses were the first manifestation in 3 cases (head and limb masses in case 7 and case 8 and 9 respectively, Figure 1), polydipsia and polyuria in 2 cases (cases 1, 10), abdominal distension in 2 cases (cases 2, 3), epilepsy in 1 case (case 5), simple rash in 1 case (case 6) and claudication in 1 case (case 4).

**Figure 2:**
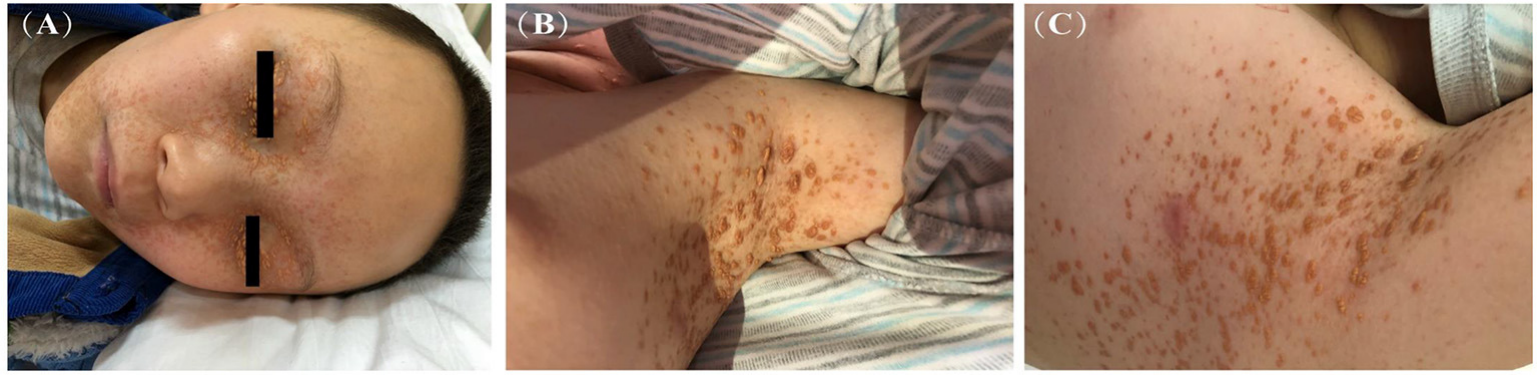
Yellow-brown papulonodular asymptomatic lesions of JXG (case 10): at the face **(A)** and left axilla **(B)** of the onset and left axilla **(C)** after treatment.

**Figure 3:**
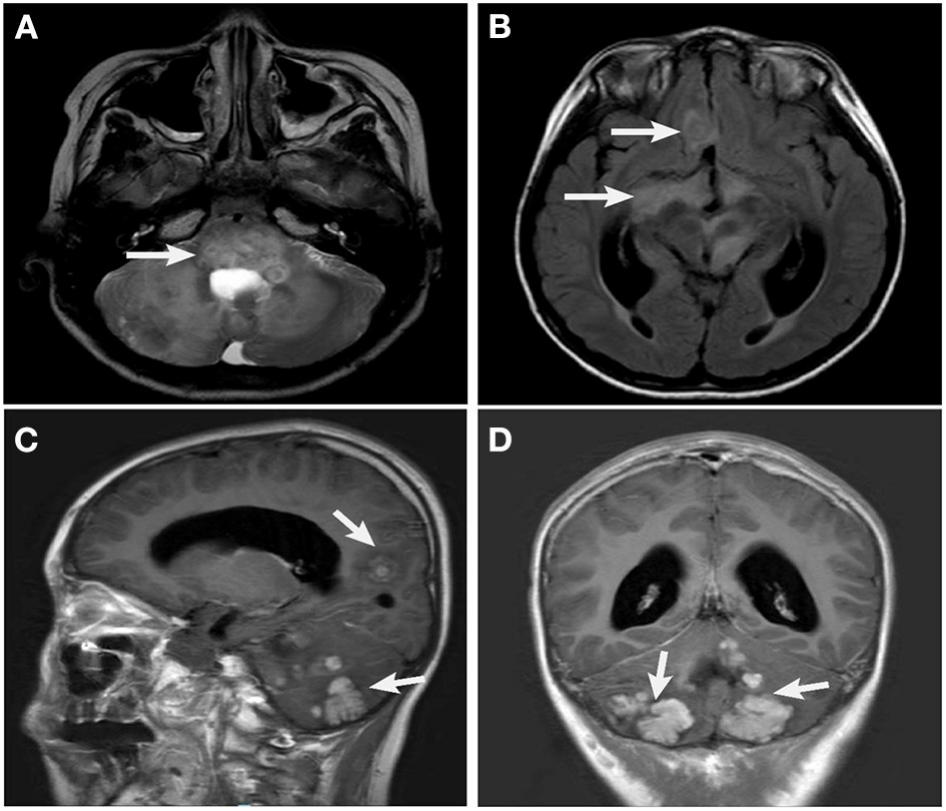
Head MRI (case 10): Brainstem **(A)**, optic pathway, sellar region, anterior middle cranial fossa **(B)** and cerebellum **(C,D)**, multiple space occupying lesions with slightly longer T2 signal, high signal intensity on FLAIR image, and uneven signal intensity of the lesion.

Skin lesions were observed in 6 cases. Most were presented as scattered red or yellow nodules with 0.5–1.0 cm in diameter (Figure 2). All patients had extracutaneous lesions including central nervous system in 6 patients [brain parenchyma, pituitary, or both brain parenchyma and pituitary in 4, 1, 1 case(s) respectively, Figure 3], lung in 5 patients, bone in 4 patients, liver in 3 patients, eye in 2 patients, endocrine system in 2 patients (such as parotid, thyroid, testis, and pancreas glands), hematological system in 2 patients and muscle in 1 patient (Table 1).

All children underwent pathogen examination, biopsy, and immunophenotyping of nucleated cells in bone marrow to exclude other diseases, such as neurofibromatosis or juvenile myelomonocytic leukemia, which are commonly accompanied by JXG.

### Pathological and Genetic Results

Clinical presentation, histopathology, and immunohistochemistry are essential for diagnosis of JXG. In three patients, diagnosis of JXG was made based on biopsy performed on lesion samples of more than two organs, including skin, bone, liver, brain tissue, muscle, testis, bone marrow (Table 1). In the other 7 patients, diagnosis of JXG was based on clinical manifestation, laboratory examination, imaging, or ophthalmic examinations.

**Figure 4:**
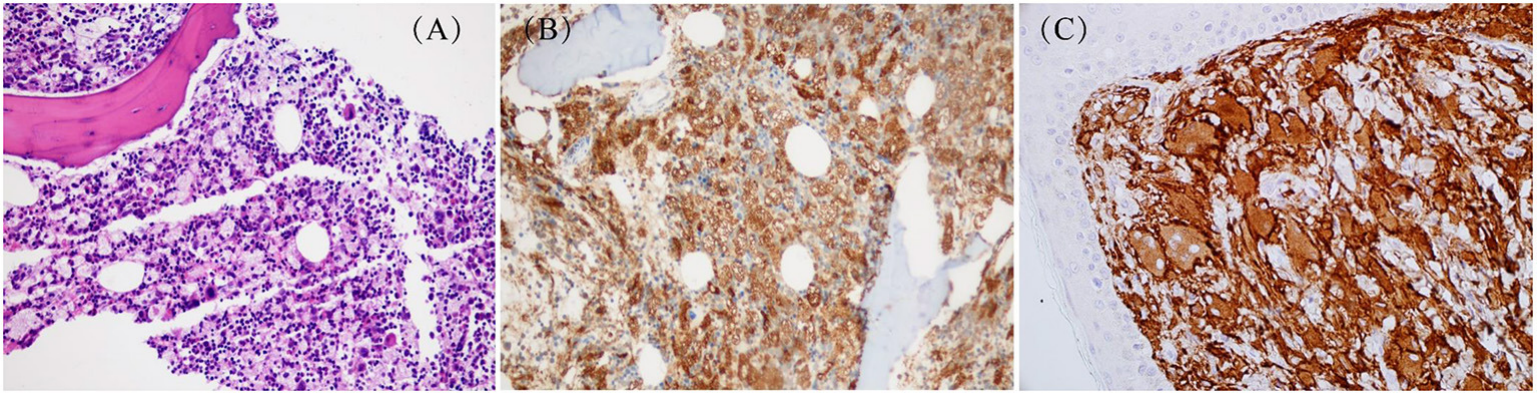
Pathological changes about skin in JXG: **(A)** HE staining: X200 (case 9); **(B)** Special staining: CD68(+)(case 9); **(C)** Special staining: CD163(+) (case 3).

Touton-type giant cells and foamy histiocytes were present in 5 and 3 cases, respectively. The immunohistochemistry results showed CD68/CD163, Fascin and Factor XIIIa positive lesional histiocytes in all 10 patients (Figure 4). S-100 positive histiocytes was present in one patient. The median value of Ki-67 was 10% (1–50%). Myeloperoxidase was positive in 8 patients. Langerin and CD1a expression were negative in all 10 patients. Furthermore, *BRAF V600E, PIK3* mutations, and ALK rearrangement were not detected in both lesion tissues and/or plasma in 7 patients. These genetic tests were not performed in the remaining 3 patients.

### Treatment and Prognosis

All the patients received chemotherapy (Table 2). Nine patients received the first-line chemotherapy after diagnosis, among them, case 10 accepted first-line chemotherapy in other hospitals for 1 year before admission to our hospital for second-line chemotherapy due to disease progression. Of the 8 children who received first-line chemotherapy in our hospital, 4 showed controlling or mitigation of their lesions. After 12 weeks of treatment, case 1 was evaluated as AD-B but lost to follow-up. Three patients (cases 4, 6, and 7) had finished the chemotherapy course and were evaluated as AD-S until the last follow-up. The other 4 patients were shifted to second-line chemotherapy due to poorly controlled diseases. The rate of response to the first-line chemotherapy was 40.0%.

**Table 2:** Treatment and prognosis.

**Pt**	**Treatment**			**Duration of follow-up (months)**	**Disease status at last follow-up**	**Permanent sequelae**
	**First-line (weeks)**	**Second-line (cycles)**	**Other treatment**			
1	12	-	-	106	AD-B	Central diabetes insipidus
2	6	2B	-	70	AD-S	-
3	2	1B	-	3	Dead	-
4	25^*^	-	-	23	AD-B	-
5	5	4B + 2A	-	21	AD-S	-
6	52^*^	-	-	19	AD-B	-
7	25^*^	-	-	9	AD-B	-
8	-	1A	Liver transplantation	9	AD-B	Liver cirrhosis, liver failure
9	12	3B	-	9	AD-B	-
10	52^#^	4B + 4A^*^	-	28	AD-B	Movement disorders, central diabetes insipidus, diminution of vision, abnormal thyroid dysfunction

*Pt, patient; A, Cytarabine + VDS + Dexamethasone; B, Cytarabine + VDS + Dexamethasone + Cladribine; AD-S, AD-Stable; AD-B, AD-Better*.

* *Treatment course finished*.

# *This patient accepted first-line treatment in other hospitals for 1 year*.

Six children were treated with second-line therapy. In one patient (case 8), arm A regimen (cytarabine + VDS + dexamethasone) was directly administered as the initial treatment after diagnosis due to severe condition and involvement of multiple organs. He underwent liver transplantation after one course of chemotherapy due to liver involvement and serious complications (liver cirrhosis and liver failure) and is now alive, 15 months after transplantation. In the other 5 patients, three were evaluated as AD-I (cases 2, 3, 9); two were in AD-W and AD-S due to developed limp (case 10) or unimprovement in lung involvement (case 5), respectively. It was noteworthy that these five patients were all treated with arm B regimen (cytarabine + VDS + dexamethasone + cladribine). During second-line chemotherapy, case 3 died of severe pulmonary infection and respiratory failure resulting from myelosuppression. Case 2 was evaluated as AD-S after 2 cycles of treatment, however, this patient was lost to follow-up. The conditions of the other 3 patients were improved after second-line chemotherapy, and 1 patient (case 10) had finished the course of chemotherapy and survived without events for 6 months. The rate of response to the second-line chemotherapy was 66.7%.

**Figure 5:**
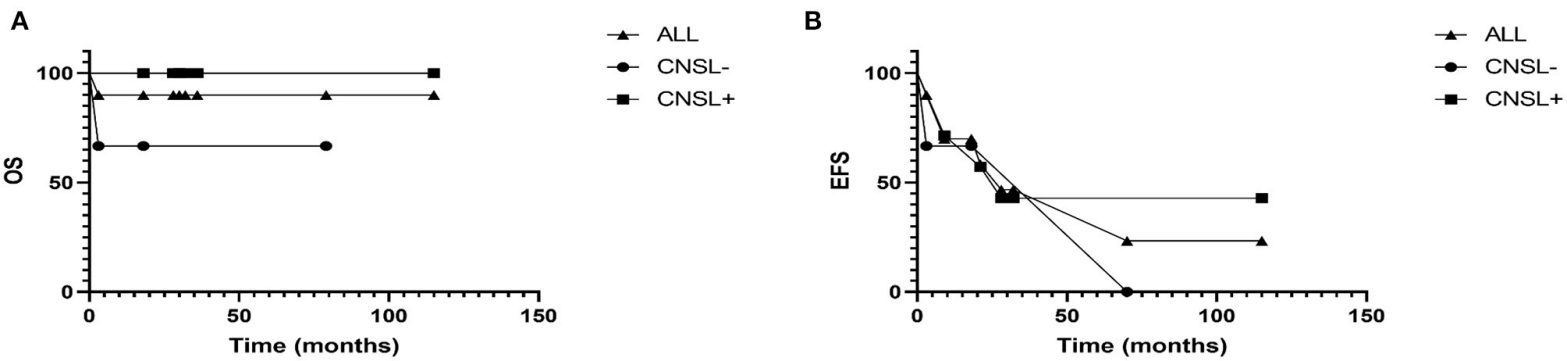
**(A)** The overall survival rate of patients with and without central nervous system were 100.0 and 75.0%, respectively. **(B)** And the 2-year event-free survival rate of patients with and without central nervous system were 50.0 and 75.0%, respectively.

The patients were followed-up through March 31, 2021. The median follow-up time was 29 (3–115) months. By the end of follow-up, 1 patient (case 3) died, and 1 patient (case 2) was lost to follow-up in AD-S. Among the 8 patients survived, two and six patients were evaluated as AD-S and AD-B, respectively. Three had permanent sequelae. Case 1 had persistent central diabetes insipidus; case 8 had liver cirrhosis; case 10 had movement disorders, central diabetes insipidus, diminution of vision and thyroid dysfunction. The 2-year OS and EFS of patients with and without central nervous system involvement were 100.0 and 75.0%, 50.0 and 75.0%, respectively (Figure 5). Due to the small sample size, the impact of central nervous system involvement on prognosis of the patients was not analyzed statistically.

## Discussion

JXG is a kind of non-Langerhans cell histiocytosis characterized by yellow papules in the skin. JXG typically occurs in infancy or early childhood, and the typical clinical manifestation is single or multiple yellowish-brown solid papules or nodules (1, 2), which mostly occur in face, neck, and upper torso. Although skin involvement is the only manifestation in most cases of JXG, involvements in other systems, namely systemic JXG which is very rare, can also be observed (9). JXG has been reported to associate with neurofibromatosis-1 (NF1) and juvenile myelomonocytic leukemia (JMML) (1).

In this study, we described clinical characteristics and treatment of 10 patients with systemic JXG, including 7 patients with intracranial lesions. This retrospective study showed that the patients with systemic JXG were mostly boys (males to females = 4:1), which is consistent with previous reports (10, 11). In terms of the age of onset, it was previously reported that 15–20% of patients had lesions at birth and that more than 75% had lesions within their first year of life (12). However, our study found that only 1 patient with systemic JXG was <1 year old, children younger than 2 years old accounted for 50%, and 2 patients were older than 6 years. Thus, it suggests that systemic JXG could occur in older children, but most of them were under 2 years old. Involvement in central nervous system, especially pituitary gland, was the second most common lesion in systemic JXG, only less than that in skin. It was reported that the onset age of patients with intracranial lesions was older than that of patients without intracranial lesions (11). However, there was no difference in the age of onset in our study, which might be due to the small sample size. In addition, this study showed a long interval from onset to diagnosis in children with central diabetes insipidus. It suggests that in the early stage of the disease, the clinical manifestations may be atypical, resulting in delayed diagnosis and permanent sequelae.

The pathological features of JXG include clear boundary of the lesions, dense infiltration of histiocytes in the lesion tissues, and scattering of lymphocytes, plasma cells, and eosinophils. Foam cells, foreign body giant cells, and Touton giant cells could be seen in the mature stage of the disease. Immunohistochemistry showed positive staining for Factor XIIIa, CD68, CD163, vimentin, anti-CD4 and negative staining for Langerin and CD1a (12, 13). In our study, the specific immunohistochemical markers CD68/CD163 and Factor XIIIa were all positive. However, Touton giant cells was observed in only 50% of our patients, much lower than that in previous report in which typical Touton giant cells were found in 85% of cases (12). Therefore, the diagnosis of JXG should be based on immunohistochemistry.

LCH, JXG, and ECD are all histiocytic disorders. The difference between LCH and JXG lies in immunohistochemistry. The LCH cells were positive staining for Langerin or CD1a (14). In a previous study, some JXG patients carried *BRAF V600E* mutations (3, 15). However, according to the new WHO classification criteria, the family of JXG with ERK-pathway mutations are now classified within the “L” (Langerhans) group, which includes LCH and ECD (16). However, there do exist some conflicts in the diagnostic criteria. To solve these problems, Picarsic et al. (17) proposed a revised diagnostic algorithm to distinguish between CNS-JXG and pediatric ECD, which included an initial morphologic diagnosis with a final integrated diagnosis after clinical-radiographic and molecular correlation. They advised long-term follow-up to determine if pediatric BRAF V600E positive CNS-JXG is a distinct entity in the L-group histiocytosis category or represents an expanded pediatric spectrum of ECD. However, patients with CNS-JXG in our study did not carry BRAF V600E mutation, including one patient who underwent brain biopsy.

Most skin lesions in children with JXG are self-healing and would disappear within 3–6 years without treatment. On the other hand, there is no generally accepted chemotherapy protocol in treatment of systemic JXG. Current treatment regimens include surgery, chemotherapy, radiotherapy, and immunosuppressant therapy (18). In addition, hematopoietic stem cell transplantation was used to treat refractory extracutaneous JXG in some studies (19, 20). Both LCH and JXG are histiocytic diseases and characterized by abnormal proliferation of dendritic cells. Previous reports showed that the regimen for treating LCH (corticosteroids and vincristine) could bring quick relief to patients with multisystem JXG (21, 22). In addition, cladribine combined with cytarabine showed obvious effectiveness of treating patients with multisystem JXG (23, 24). Therefore, we used VDS + prednisone as the first-line chemotherapy and cytarabine + VDS + dexamethasone ± cladribine as the second-line chemotherapy. By the end of follow-up, two patients were evaluated as AD-S, and six patients were evaluated as AD-B. The rates of response to the first-line and second-line chemotherapy were 40 and 66.7%, respectively. Thus, the effectiveness of first-line chemotherapy of JXG patients with multisystem involvements was inadequate, and more than half of the patients needed second-line chemotherapy for disease control. This study also showed that the intense second-line chemotherapy was tolerable to pediatric patients, only 1 patient died of severe infection resulting from myelosuppression.

The prognosis of patients with systemic JXG was good in previous study, 75% of children could obtain long-term disease-free survival, and <10% of children may die (23, 24). In our study, 9 of 10 children survived, only one died of chemotherapy-related complications. Intracranial lesions did not affect the prognosis of children with systemic JXG, although may leave permanent sequelae such as central diabetes insipidus. In addition, eye involvement may also leave permanent sequelae such as diminution of vision.

## Conclusion

In this study, the clinical manifestations of the children with systemic JXG were diverse and commonly involved in the central nervous system. The chemotherapy protocol for LCH may be effective for patients with systemic JXG. Central nervous system involvement may not impact overall survival, although may result in serious permanent sequelae. Considering the small sample size and lack of analysis on risk-related factors, it is still necessary to confirm the efficacy of LCH chemotherapy and related adverse reactions in studies with a large cohort of systemic JXG patients and a long-term follow-up.

## Data Availability Statement

The original contributions presented in the study are included in the article/supplementary material, further inquiries can be directed to the corresponding authors.

## Ethics Statement

This study was conducted in accordance with the Declaration of Helsinki and approved by the Institutional Review Board (IRB) of Beijing Children's Hospital, Capital Medical University (2020-z-134). All parents signed informed consent forms.

## Author Contributions

AW, HL, and LH: writing—original draft. LZ, YG, and ZL: writing—review and editing. YY and QZ: data curation. HM and DW: formal analysis. ZL, TW, and RZ: project administration. All authors contributed to the article and approved the submitted version.

## Conflict of Interest

The authors declare that the research was conducted in the absence of any commercial or financial relationships that could be construed as a potential conflict of interest.

## Abbreviations

JXG: juvenile Xanthogranuloma
LCH: Langerhans Cell Histiocytosis
VDS: vindesine
6-MP: 6- mercaptopurine
Ara-c: cytosine arabinoside
2CDA: 2-Chlorodeoxyadenosine
NAD: Non-active Disease
AD: Active Disease
AD-B: AD-Better
AD-I: AD-Intermediate
AD-S: AD-Stable
AD-W: AD-Worse
ECD: Erdheim-Chester Disease.
